# Jet-Setting Koalas Spread Cryptococcus gattii VGII in Australia

**DOI:** 10.1128/mSphere.00216-19

**Published:** 2019-06-05

**Authors:** Laura J. Schmertmann, Patrizia Danesi, Juan Monroy-Nieto, Jolene Bowers, David M. Engelthaler, Richard Malik, Wieland Meyer, Mark B. Krockenberger

**Affiliations:** a Sydney School of Veterinary Science, The University of Sydney, Sydney, New South Wales, Australia; b The Westmead Institute for Medical Research, Westmead, New South Wales, Australia; c Molecular Mycology Research Laboratory, Centre for Infectious Diseases and Microbiology, The University of Sydney—Westmead Clinical School, Faculty of Medicine and Health, Sydney, New South Wales, Australia; d Istituto Zooprofilattico Sperimentale delle Venezie, Legnaro, Padua, Italy; e Translational Genomics Research Institute, Flagstaff, Arizona, USA; f Centre for Veterinary Education, The University of Sydney, Sydney, New South Wales, Australia; g Marie Bashir Institute for Infectious Diseases and Biosecurity, The University of Sydney, Sydney, New South Wales, Australia; h Veterinary Pathology Diagnostic Services, The University of Sydney, Sydney, New South Wales, Australia; Duke University Medical Center

**Keywords:** cryptococcosis, *Cryptococcus*, koala, molecular epidemiology, veterinary microbiology

## Abstract

Cryptococcus gattii molecular type VGII is one of the etiologic agents of cryptococcosis, a systemic mycosis affecting a wide range of host species. Koalas (Phascolarctos cinereus) exhibit a comparatively high prevalence of cryptococcosis (clinical and subclinical) and nasal colonization, particularly in captivity. In Australia, disease associated with C. gattii VGII is typically confined to Western Australia and the Northern Territory (with sporadic cases reported in eastern Australia), occupying an enigmatic ecologic niche. A cluster of cryptococcosis in captive koalas in eastern Australia (five confirmed cases, a further two suspected), caused predominantly by C. gattii VGII, was investigated by surveying for subclinical disease, culturing koala nasal swabs and environmental samples, and genotyping cryptococcal isolates. *URA5* restriction fragment length polymorphism analysis, multilocus sequence typing (MLST), and whole-genome sequencing (WGS) provided supportive evidence that the transfer of koalas from Western Australia and subsequently between several facilities in Queensland spread VGII into uncontaminated environments and environments in which C. gattii VGI was endemic. MLST identified VGII isolates as predominantly sequence type 7, while WGS further confirmed a limited genomic diversity and revealed a basal relationship with isolates from Western Australia. We hypothesize that this represents a founder effect following the introduction of a koala from Western Australia. Our findings suggest a possible competitive advantage for C. gattii VGII over VGI in the context of this captive koala environment. The ability of koalas to seed C. gattii VGII into new environments has implications for the management of captive populations and movements of koalas between zoos.

**IMPORTANCE** Cryptococcus gattii molecular type VGII is one of the causes of cryptococcosis, a severe fungal disease that is acquired from the environment and affects many host species (including humans and koalas). In Australia, disease caused by C. gattii VGII is largely confined to western and central northern parts of the country, with sporadic cases reported in eastern Australia. We investigated an unusual case cluster of cryptococcosis, caused predominantly by C. gattii VGII, in a group of captive koalas in eastern Australia. This research identified that the movements of koalas between wildlife parks, including an initial transfer of a koala from Western Australia, introduced and subsequently spread C. gattii VGII in this captive environment. The spread of this pathogen by koalas could also impact other species, and these findings are significant in the implications they have for the management of koala transfers and captive environments.

## Abstract (precis)

Cryptococcus gattii molecular type VGII is one of the causes of cryptococcosis, a severe fungal disease that is acquired from the environment and affects many host species (including humans and koalas). In Australia, disease caused by C. gattii VGII is largely confined to western and central northern parts of the country, with sporadic cases reported in eastern Australia. We investigated an unusual case cluster of cryptococcosis, caused predominantly by C. gattii VGII, in a group of captive koalas in eastern Australia. This research identified that the movements of koalas between wildlife parks, including an initial transfer of a koala from Western Australia, introduced and subsequently spread C. gattii VGII in this captive environment. The spread of this pathogen by koalas could also impact other species, and these findings are significant in the implications they have for the management of koala transfers and captive environments.

## INTRODUCTION

The Cryptococcus gattii species complex comprises a group of environmentally acquired fungal pathogens that, along with the Cryptococcus neoformans species complex, are the etiologic agents of cryptococcosis (1, 2). A proposal to divide these species complexes into seven distinct species (according to genotypic differences) (3) remains under debate within the cryptococcal research community and has recently been reported as premature (4). Henceforth, the terms C. gattii and C. neoformans will be used to refer to their respective species complexes, with restriction fragment length polymorphism (RFLP) analysis molecular types (VGI to VGIV and VNI to VNIV) also used, when known. This disease affects a wide range of host species, from mammals to birds and reptiles (5). In Australia, the C. gattii population comprises mostly C. gattii VGI and VGII, with VGII largely confined to Western Australia (WA) and the Northern Territory (NT) (1, 6–8). Interestingly, C. gattii VGII has been implicated in several case clusters and outbreaks in various host species across several continents (most notably, Vancouver Island, Canada) (9–12), while C. gattii VGI disease tends to be more sporadic within a range of endemicity (although some outbreaks in animals have been reported) (5, 13, 14).

Koalas (Phascolarctos cinereus) appear particularly prone to developing cryptococcosis caused by C. gattii, with clinical disease, asymptomatic antigenemia (subclinical disease), and nasal colonization well documented among captive individuals (5, 15–19) and free-ranging populations (20, 21). This may in part be related to the preferred habitat and diet of the koala, eucalypt trees, also exhibiting a strong association with C. gattii VGI as an ecologic niche (12, 22–24). Seemingly healthy koalas are able to carry C. gattii VGI and VGII with them when translocated within Australia or internationally, either through colonization of the sinonasal mucosa or within constrained foci of infection (subclinical disease) (5, 25–27). Other animals can also carry C. gattii and C. neoformans in the nasal cavity and gastrointestinal tract, and there has been speculation that this presents a possible method of dispersal (28–34), especially in relation to birds. A prior study suggested that koalas may also seed new environments with C. gattii but did not genotype isolates or include confirmatory molecular evidence (16).

**FIG 1:**
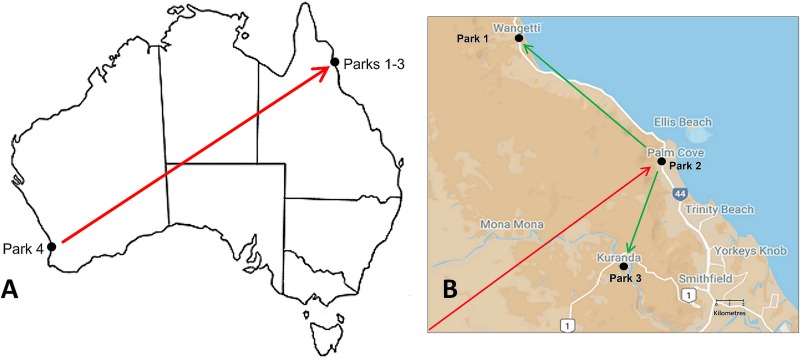
(A) Map of Australia showing the approximate location of the four wildlife parks (parks 1 to 4) studied. The red arrow indicates the movement of a koala from park 4 to park 2 approximately 10 years prior to this study. (B) Map showing the location of parks 1 to 3 within the Cairns region of Queensland, Australia. Green arrows indicate the movement of 20 koalas from park 2 to parks 1 and 3 in 2016 (approximately 10 koalas to each park).

A case cluster of cryptococcosis largely attributed to C. gattii VGII was observed in captive koalas in eastern Australia, with five confirmed clinical cases over a 4-year period, including three mortalities (Table 1), and a further two suspected cases (based on cryptococcal antigen titers near the time of death). No known cases of cryptococcosis had occurred in any animals housed at these facilities prior to 2013. These koalas were regularly moved between three co-owned wildlife parks in Queensland, Australia (parks 1, 2, and 3), and an individual had previously been introduced into this population from a location in Western Australia (park 4) (Fig. 1). The involvement of C. gattii VGII prompted concerns regarding the emergence of this molecular type in eastern Australia and the implications this may have for managing the case cluster in these koalas (given that VGII may be less susceptible to fluconazole [35]). This scenario provided an opportunity to further characterize cryptococcosis in captive koalas while also observing the potential for koalas to translocate C. gattii into new environments. The aim of this study was to characterize the fine-scale molecular epidemiology of this case cluster by (i) determining the environmental burden of C. gattii and the prevalence of cryptococcal nasal colonization in the koalas housed at parks 1 to 3, (ii) using molecular epidemiologic tools to characterize environmental, colonizing, and disease strains from parks 1 to 4, and (iii) assessing the prevalence of subclinical disease (asymptomatic antigenemia) among the koalas domiciled in parks 1 to 3.

**TABLE 1:** Koalas (*n* = 5) at co-owned captive facilities in the Cairns region of Queensland, Australia, with confirmed clinical cryptococcosis attributed to infection with the Cryptococcus gattii species complex from 2013 to 2016

Koala	Year	Antigenemia (LCAT titer)^*a*^	Primary lesion^*b*^	Molecular type	Outcome
1	2013	U^*c*^	Thoracic	VGIIb	Death
2	2014	+ (1:256)	Nasal	VGIIb	Death
3	2014	+^*d*^	CNS	VGI	Death
4	2016	+ (1:128)	Inguinal LN	VGIIb	Survival
5	2016	+ (1:512)	Nasal	VGIIb	Survival

a LCAT, latex cryptococcal antigen agglutination test.

b CNS, central nervous system; LN, lymph node.

c U, unknown.

d LCAT titer unavailable, cryptococcal antigen lateral flow immunoassay positive.

## RESULTS

### Koala nasal swabs.

Cryptococcal nasal colonization was identified in 14 of 44 (32%) koalas on at least one occasion (Table 2). Of the colonized koalas, 9/14 were tested twice, with 5/9 progressing from negative to positive on nasal swab culture, 3/9 remaining consistently positive, and 1/9 progressing from positive to negative (Table 2). This resulted in a total of 17 positive culture result events among the 14 koalas. Low, moderate, and heavy cryptococcal colonization burdens were reported on five, three, and nine occasions, respectively. Among the three consistently positive koalas, one exhibited a heavy cryptococcal burden on both occasions, while the other two progressed from moderate to heavy.

**TABLE 2:** Koalas (*n* = 14), ordered by identification number, at three co-owned captive facilities in the Cairns region of Queensland, Australia, that tested positive for Cryptococcus gattii species complex nasal colonization on at least one of two time points

Koala number	2015		2017		Antigenemia (titer)^*c*^
	Park^*a*^	Nasal colonization^*b*^ (molecular type)	Park^*a*^	Nasal colonization^*b*^ (molecular type)	
LS40	1	+ (VGIIb)	1	NT^*d*^	+ (1:8)
LS49	2	+++ (VGI)	3	+++ (VGI)	+^*e*^
LS55	2	++ (VGIIb)	3	NT	+ (1:512)
LS56	2	++ (VGIIb)	3	+++ (VGI, VGIIb)	+ (1:8)
LS67	3	+++ (VGI)	3	NT	−
LS69	3	+++ (VGI)	3	NT	+ (1:8)
LS73	3	+ (VGI)	3	−	−
LS75	3	+ (VGI)	3	NT	−
LS77	3	++^*f*^	3	+++ (VGI)	+ (1:4)
LS291	2	−	1	+^*f*^	−
LS292	2	−	1	+++ (VGI)	−
LS298	2	−	1	+ (VGIIb)	−
LS307	2	−	3	+++ (VGIIb)	+^*e*^
LS311	2	−	3	+++ (VGI, VGIIb)	−

a Park 1 is located at 16°39′47.1′′S 145°33′51.9′′E, park 2 at 16°45′28.9′′S 145°39′46.4′′E, and park 3 at 16°49'′07.9′′S 145°37′58.3′′E (see Fig. 1).

b +, low degree of cryptococcal growth (1 to 10 colonies); ++, moderate (11 to 100 colonies); +++, heavy (>100 colonies).

c Antigenemia results reflect if these koalas tested positive (+) at any time between 2014 and 2018 and the highest recorded latex agglutination cryptococcal antigen test titer.

d NT, not tested.

e Lateral flow immunoassay only.

f No isolate available.

In 2015, 14% (1/7), 14% (3/22), and 45% (5/11) of koalas were positive for nasal colonization at parks 1, 2, and 3, respectively. The single positive individual located at park 1 had recently been transferred from park 2. In 2017, following the closure of park 2 and transfer of all koalas to parks 1 and 3, 19% (3/16) and 33% (5/15) of koalas were positive at parks 1 and 3, respectively.

### Environmental samples.

**FIG 2:**
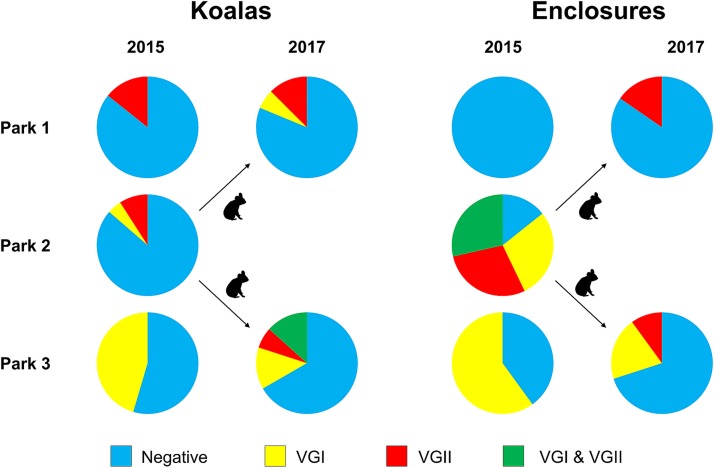
Proportions of koalas and enclosures colonized by members of the Cryptococcus gattii species complex across three wildlife facilities (parks 1 to 3) on two sampling occasions, 2015 and 2017. Blue indicates negative koalas/enclosures (not colonized), while green, red, and yellow indicate koalas or enclosures colonized by C. gattii VGI, VGII, or both VGI and VGII, respectively. Park 2 was not sampled in 2017 due to its closure; arrows indicate the movement of koalas from park 2 to parks 1 and 3 in 2016.

In 2015, 56% (9/16) of all enclosures cultured positive for *Cryptococcus* spp. (colonies exhibiting the brown color effect were observed). At park 2, 86% (6/7) of enclosures were positive, while at park 3, 60% (3/5) were positive (Fig. 2). No enclosures at park 1 cultured positive for *Cryptococcus* spp. in 2015 (Table 3). Of the nine positive enclosures, six had heavy and three had low cryptococcal burdens. An isolate could not be obtained from one of the low-positive enclosures at park 3 due to overgrowth of filamentous fungi on the culture plate.

**TABLE 3:** Environmental sampling results for *Cryptococcus* spp., using bird seed agar culture and *URA5* restriction fragment length polymorphism typing, at three co-owned wildlife parks in the Cairns region of Queensland, Australia, across two sampling occasions

Park^*a*^	Year	No. of enclosures	No. (%) of enclosures culture positive for *Cryptococcus* spp.	C. gattii molecular type(s) identified (no. of enclosures)
1	2015	4	0	NA^*b*^
	2017	13	2 (15)	VGII (2)
2	2015	7	6 (86)	VGI (2); VGII (2); both VGI and VGII (2)
3	2015	5	3 (60)	VGI (3)
	2017	10	3 (30)	VGI (2); VGII (1)

a Park 2 closed in 2016, and parks 1 and 3 subsequently expanded (increased the number of enclosures) to accommodate koalas from park 2.

b NA, not applicable.

In 2017, 22% (5/23) of all enclosures tested positive for *Cryptococcus* spp., comprising two enclosures at park 1 and three at park 3. This meant that at parks 1 and 3, *Cryptococcus* spp. were cultured from 15% (2/13) and 30% (3/10) of enclosures, respectively (Table 3). The degree of cryptococcal growth was classified as low for two positive enclosures, moderate for two, and heavy for one. Isolates were obtained from all five positive enclosures. The change in enclosure numbers from 2015 (16) to 2017 (23) was due to the expansion of parks 1 and 3 (and sometimes the division of previously larger enclosures into several small enclosures) due to the transfer of all koalas from park 2.

Cryptococcal antigenemia was detected in 20 of 58 (34%) koalas on at least one occasion during this study, with two testing positive by a lateral flow assay (LFA) only (including one case where a confirmatory latex cryptococcal antigen agglutination test [LCAT] could not be run due to insufficient sample), and the remaining 18 were positive using both tests (36). Confirmed clinical cases accounted for four koalas (with LCAT titers of 1:128, 1:256, 1:512, and one unknown). A further two were presumptive clinical cases, based on LCAT titers of 1:128 in both cases and their sudden deaths. Thus, six antigen-positive cases (10% of the 58 koalas sampled) were symptomatic. The remaining 14 exhibited asymptomatic cryptococcal antigenemia, resulting in a subclinical disease prevalence of 24% (14/58). One koala (koala 1) (Table 1) with confirmed clinical cryptococcosis (based on postmortem findings) was not tested for antigenemia. Among the 14 koalas with subclinical cryptococcosis, two were LFA positive only, while the remaining 12 returned positive LCAT results with titers of 1:2 (3/12), 1:4 (3/12), 1:8 (3/12), and 1:16 (3/12).

Nasal colonization results were available from 16/20 koalas with cryptococcal antigenemia (excluding two confirmed clinical cases, one suspected case, and one other individual). Of these 16 koalas, 9 were negative for nasal colonization on all occasions tested, while 7 were positive on at least one occasion (Table 2).

### Molecular and mating type determination.

A total of 71 *Cryptococcus* species strains were obtained, with 5 disease-associated, 33 nasal colonizing, and 33 environmental isolates. C. gattii VGI accounted for 39/71 isolates (one disease associated, 21 nasal colonizing, and 17 environmental). C. gattii VGI was not isolated from any samples collected (nasal or environmental swabs) at park 1 (Fig. 2). C. gattii VGII accounted for the remaining 32/71 isolates (4 disease associated, 12 nasal colonizing, and 16 environmental). All 32 C. gattii VGII isolates from parks 1 to 3 were determined to be mating type α.

At park 1, in 2015, two C. gattii VGII isolates (of the same sequence type [ST]) were obtained from a single nasal swab from a koala recently transferred from park 2. In 2017, seven isolates were obtained, with C. gattii VGI accounting for two nasal colonizing isolates from one koala and C. gattii VGII accounting for one nasal colonizing isolate and four environmental isolates. Both colonized koalas had been transferred from park 2.

At park 2, one disease isolate was identified as C. gattii VGII. Three koalas were colonized by C. gattii, with C. gattii VGI in one and VGII in the remaining two. Among the six positive enclosures at this park, 2/6 contained only C. gattii VGII, 2/6 contained only C. gattii VGI, and the remaining 2/6 had a mixed burden of both C. gattii VGI and VGII (Fig. 2).

At park 3, C. gattii VGII accounted for 3/4 disease cases (all three had a recent history of previously residing at park 2), while C. gattii VGI was identified as the etiologic agent in a single case. All isolates collected from park 3 in 2015 were identified as C. gattii VGI—this included nasal-colonizing isolates from four koalas and environmental isolates from two enclosures. In 2017, five koalas were colonized by C. gattii and either exhibited a C. gattii VGI burden (2/5), a C. gattii VGII burden (1/5), or mixed colonization by both VGI and VGII (2/5). All koalas with any C. gattii VGII colonization had a history of relocation from park 2. Among the three enclosures identified as positive for C. gattii in 2017, two contained C. gattii VGI and one contained C. gattii VGII (Table 3).

A total of 34 C. gattii VGII isolates (16 environmental, 17 colonizing, 1 disease) previously collected from park 4 were identified for comparative inclusion in this study. Mating type α accounted for 33/34 isolates; a single mating type **a** environmental isolate was identified.

### Multilocus sequence typing.

Among the 32 C. gattii VGII isolates from parks 1 to 3 identified in this study, 30/32 were sequence type (ST) 7. The remaining two isolates were novel to the C. gattii multilocus sequence typing (MLST) database and were classified as ST 539; these were both nasal-colonizing isolates collected at the same time from a koala residing at park 2. Allele differences were observed only at the *URA5* locus, with two unique allele types identified (one for ST 7 and one for ST 539).

At park 4, 30/34 isolates were ST 7. The remainder were ST 38 and ST 48, accounting for one and three isolates, respectively. Allele type differences were observed at all seven MLST loci. Two unique allele types each were observed at the *GPD1, LAC1*, and *URA5*, whereas three allele types each were observed at *CAP59*, IGS1, *PLB1*, and *SOD1*.

**FIG 3:**
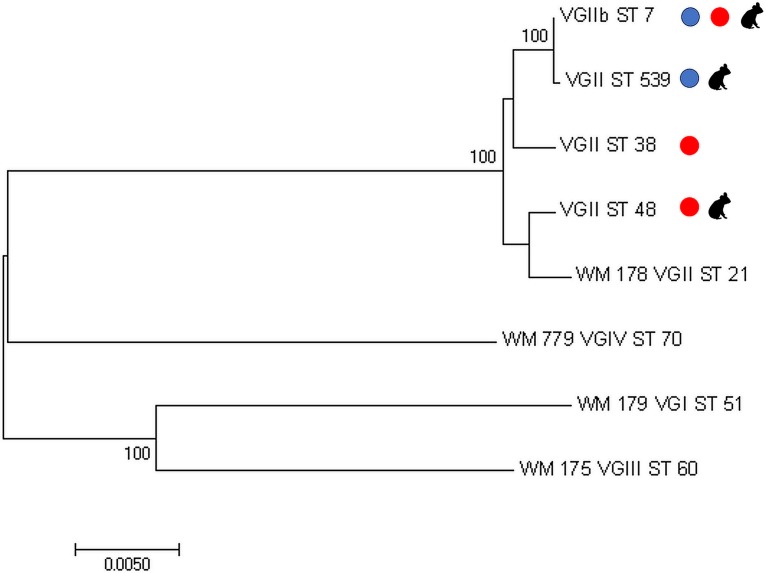
Maximum likelihood phylogenetic tree, using the Kimura 2-parameter model with gamma distribution, of the concatenated multilocus sequence typing sequences from four sequence types (STs) identified among koala disease, colonizing, and environmental isolates at three captive facilities in northern Queensland, Australia (blue circles), and a facility in Perth, Western Australia (red circles). STs found in koalas (as either disease or colonizing isolates) are identified by the koala silhouette. Cryptococcus gattii species complex standard strains (VGI, WM 179; VGII:, WM 178; VGIII, WM 175; VGIV, WM 779) were included for outgrouping. Bootstrap values were obtained from 1,000 replicates using a maximum likelihood model, with values over 70 shown. The scale bar indicates the number of nucleotide substitutions per site.

The maximum likelihood analysis of concatenated MLST sequences revealed a highly limited genetic diversity among isolates from parks 1 to 3, with ST 539 settling in the C. gattii VGIIb clade with ST 7. The isolates from park 4, however, exhibited greater diversity, with ST 48 grouping closer to the C. gattii VGII standard strain (WM 178) than to ST 7 strains (Fig. 3).

### Whole-genome sequencing.

**FIG 4:**
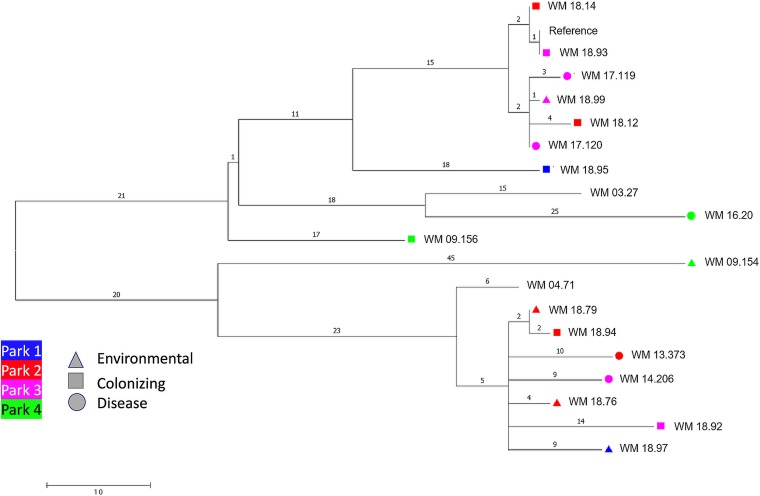
Maximum parsimony tree constructed with 95% consensus of 40 most parsimonious trees using whole-genome SNP data from 19 Cryptococcus gattii VGIIb isolates. Of these strains, 17 were isolated for the present study and 2 additional Australian isolates, WM 03.27 and WM 04.71, were included for comparison and are shown without leaf markers. The reference for SNP calling was the *de novo* assembly of WM 18.93. Colors and shapes represent the four parks and isolate type, as outlined in the key. A total of 302 high-confidence whole-genome SNP positions were considered. Polytomies represent less than 95% confidence. Branch lengths represent SNP distances.

The phylogeny of the isolates based on whole-genome single nucleotide polymorphism (SNP) data separated the isolates into two major clades, with isolates from all parks present in both clades (Fig. 4). An overall low genetic diversity is seen across the entire tree, with each clade exhibiting few differences between isolates from parks 1 to 3. In both clades, isolates from park 4 are relatively basal.

**FIG 5:**
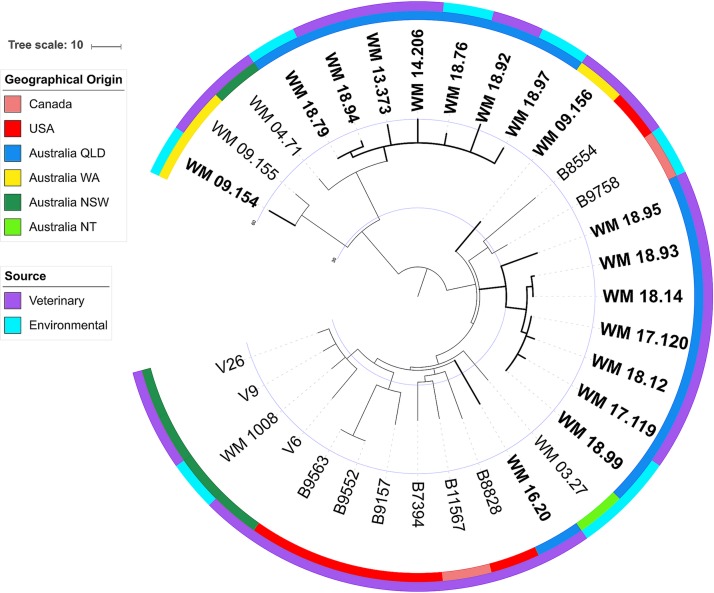
Maximum parsimony population tree for 32 environmental and veterinary isolates of Cryptococcus gattii VGIIb. Isolates from the koalas and enclosures in this study are shown in boldface font. A total of 712 high-confidence whole-genome SNP positions were considered. Outer rings display geographical origin and source of isolation. Polytomies represent consensus levels below 95%. The tree splits concordantly with Fig. 4, where the top clade represents the right split in this tree. NSW, New South Wales; NT, Northern Territory; QLD, Queensland; WA, Western Australia.

A phylogenetic tree with additional relevant genomes from veterinary and environmental isolates displays the same overall topology with two major clades of VGIIb (Fig. 5). The isolates sampled from the koalas and enclosures at parks 1 to 3 remain clustered together within their respective clades, and all koala isolates demonstrate the same relationships as in Fig. 4.

## DISCUSSION

This study provides evidence in support of the notion that koalas can seed environments with C. gattii VGII. The detection of an unusually high prevalence in an area where C. gattii VGII is considered to be nonendemic and the documented transfer of a single koala from a wildlife park in Western Australia where C. gattii VGII is endemic provided the opportunity to investigate what happens after C. gattii VGII is introduced into (i) an environment with no C. gattii (enclosures in park 1) or (ii) an environment with a moderate presence of C. gattii VGI (park 3). Our findings suggest that C. gattii VGII can indeed colonize a new environment (most likely originating from the animal transferred from park 4), be amplified and further dispersed by the presence of captive koalas, and compete successfully with an existing population of C. gattii VGI. This study also offered some insight into the effectiveness of environmental decontamination as a management tool to prevent cryptococcosis in captive scenarios.

The ability of koalas to translocate C. gattii VGII was made particularly apparent by its absence in the environment at parks 1 and 3 in 2015 and subsequent presence in 2017. This presumably was the result of the transfer of koalas from park 2, a facility with abundant environmental C. gattii VGII, to parks 1 and 3 in 2016. At park 1 in 2015, the only place we were able to detect C. gattii VGII was in the nasal cavity of one translocated koala from park 2; VGII was not detected in the environment. Although koalas were transferred between all three facilities prior to 2016, this was sporadic, typically involved just one individual at a time, and was predominantly between parks 2 and 3. Presumably by chance, this left park 1 as a low environmental presence zone for C. gattii, despite the potential amplifying effect of the captive koala population. Such low environmental presence in captive environments has been described previously (16).

In 2016, approximately 20 koalas from park 2 were transferred to parks 1 and 3, with roughly 10 individuals going to each of the two facilities, in short succession. This provided an opportunity for a mass introduction of C. gattii VGI and VGII into both environments, although park 3 already had a moderate presence of VGI. At the time, an environmental decontamination protocol was already in place at all parks. We also hypothesized that the transfer of a koala from park 4 in Western Australia, where C. gattii VGII is endemic (8), provided the initial introduction of C. gattii VGII into the environment at park 2. In further support of this hypothesis, no koalas at park 1 were positive for cryptococcal antigenemia prior to the 2016 closure of park 2 and translocation of numerous koalas, and when first sampled, no C. gattii VGI or VGII was isolated from the environment. Also, all koalas found to have C. gattii VGII disease or nasal colonization either resided at, or had a history of recent transfer from, park 2. Increased stress could play a role in this case cluster, due to concurrent transfer of large numbers of animals, but given that 3/5 confirmed cases (and one of the suspected cases) were diagnosed prior to the closure of park 2, it seems likely that this was a key site for the ongoing distribution of cases even prior to the major transfer event.

C. gattii VGII is difficult to find in natural environments in Australia, even in areas of endemicity (Western Australia and the Northern Territory) (12, 26, 37). This is in contrast with C. gattii VGI, which can be isolated with relative ease from well-established tree hollows in an ever-expanding range of Australian tree species (12, 16, 20, 23, 24, 26, 38). The occasional isolation of *C gattii* VGII from cats with cryptococcosis in eastern Australia (and no travel history) (7) suggests that it must also be present in this environment, but successful isolations are rare (39). It therefore remains a possibility that the environmental C. gattii VGII in park 2 was already present and not introduced by a koala from Western Australia. Airborne dispersal of C. gattii VGII from external sources leading to colonization of the environment in park 3 is also theoretically possible, as hypothesized in a prior study (16). Given the apparent rarity of C. gattii VGII in the environment in eastern Australia, however, both scenarios seem unlikely.

MLST analysis showed that the C. gattii VGII population in these three facilities is almost exclusively ST 7, which was the most common type found in a prior study of Australian isolates (8). Greater genetic diversity is often seen in southwestern Western Australia, where C. gattii VGII is endemic, but ST 7 is still predominant (8, 26). Therefore, our finding of a mostly ST 7 population with a highly limited diversity (based on both MLST and whole-genome sequencing [WGS]) in the three eastern Australian facilities supports the hypothesis of a founder effect from the introduction of a colonized koala arriving from Western Australia. This seems more likely when considering that the strains of park 4 in Western Australia share an ancestor with the remainder of the sampled isolates from which they diverge early on, while the isolates from the rest of the sites remained closely related according to WGS.

The recent evolutionary histories of the two clades seen in Fig. 4 appear to be dissimilar. The top clade has splits with ≥95% consensus, indicating a more gradual formation of its population structure, whereas the polytomy of the bottom clade indicates a more radial spread of the fungus from this lineage. The inclusion of two other unrelated isolates (WM 03.27 and WM 04.71) for comparative purposes in the phylogenetic tree has also suggested a relative overall clonality for C. gattii VGIIb in Australia. In relation to the global population of VGIIb, Fig. 4 and 5 reflect the same topology as described in previous studies (40–42).

These results support a prior study that showed enclosures previously culturing negative for C. gattii could become positive after the introduction of koalas with cryptococcal colonization (skin or nasal) (16). The same study found that koalas appeared able to amplify the burden of cryptococcal environmental contamination in captive environments (16). The exact mechanism of environmental seeding is unknown, but it is hypothesized here that C. gattii could either be transferred directly to the enclosure environment from the skin of the koala (particularly through colonization of the feet and nail beds [16]) or from the sinonasal cavity by sneezing or snorting during vocalization. The amplification of environmental C. gattii in captivity is thought to be related to the scarification of enclosure “furniture” combined with regular soiling by urine and fecal material, providing an optimal high creatinine substrate for cryptococcal growth (5).

Given the evidence that C. gattii may be dispersed by anthropogenic disturbances (and has been detected on footwear and car wheels in Vancouver Island, Canada) (43), one factor that should also be considered is the possible involvement of fomites in this scenario. This is particularly relevant to the initial single koala transferred from park 4 to park 2, given that captive koalas are transported with fresh eucalyptus leaves as feed (referred to as leaf browse) for the journey. This material could theoretically have also provided the introduction of C. gattii VGII into the environment, but we consider this unlikely, given that fresh eucalyptus leaves are typically not heavily contaminated by C. gattii (even in environments where koalas and their enclosure furniture are heavily colonized) (16).

Environmental decontamination as a means of managing koala cryptococcosis has long been recommended (5), but its effectiveness remains largely anecdotal. A prior study found that quaternary ammonium compounds effectively killed all C. neoformans yeasts in pigeon droppings within 30 min of contact time at a concentration of 0.062%, while rapid killing was observed at a concentration of 0.5% (44). At the facilities in this study, a concentration of 0.4% was typically used, with a 30-min contact period. However, it is unclear if this concentration is also effective against C. gattii. In the present study, the nasal colonization rate at park 3 decreased from 45% to 33% and the environmental contamination rate decreased from 60% to 30% of enclosures between 2015 and 2017 (Fig. 2), which suggests that the protocol implemented was effective. Further targeted and systematic environmental decontamination studies are required to draw conclusions about the usefulness of this as a management tool to reduce the prevalence of cryptococcosis in captive koala groups.

The current Australian requirement for koalas prior to travel or export is to test only for cryptococcal antigenemia (45). In the event of a positive result, further testing should be performed in an attempt to locate lesions and treatment should be considered, depending on the antigen titer and its persistence (21, 46). The present study brings into question whether koalas should also be tested for nasal and/or skin colonization prior to travel to attempt to prevent the introduction of novel C. gattii genotypes into new environments and/or to guide management practices at their destination. This would be hampered, however, by the unknown sensitivity and detection limit of nasal swabbing as a means of determining colonization status and by the difficulty in clearing nasal colonization with current treatments (19). Thus, preventing colonized koalas from seeding new environments with C. gattii is likely not feasible, but this possibility remains an important factor to consider in some scenarios (particularly when obtaining new koalas from areas of C. gattii VGII endemicity).

This study offers valuable insights into the management of captive koala cryptococcosis and the composition of the C. gattii VGII population in eastern Australia. The relative environmental competitiveness of C. gattii VGI and VGII remains uncertain, but our findings could suggest an increased fitness of VGII in this scenario. Confirmation of the capacity of koalas to translocate C. gattii to previously uncontaminated environments, and provision of compelling new evidence that this extends to C. gattii VGII, has implications for the prevention and management of potential outbreaks in Australia.

## MATERIALS AND METHODS

### Locations.

All samples originated from four captive animal facilities in Australia: three related wildlife parks (under the same ownership) in the vicinity of Cairns, Queensland (park 1, 16°39′47.1′′S 145°33′51.9′′E; park 2, 16°45′28.9′′S 145°39′46.4′′E; and park 3, 16°49′07.9′′S 145°37′58.3′′E) and one near Perth, WA (park 4, 31°50′03.6′′S 115°57′01.2′′E) (Fig. 1). Koalas were regularly transferred between parks 1 to 3. Environmental samples and nasal swabs were collected from parks 1 to 3 on two occasions: December 2015 and September 2017. Four of the koalas with clinical cryptococcosis were diagnosed while domiciled at park 3 (koalas 1, 2, 4, and 5) (Table 1), with all four having a history of recent transfer from park 2. The fifth koala was diagnosed while residing in park 2 and had been located there for several years. A male koala from park 4 was translocated to park 2 approximately 10 years prior to this study (Fig. 1), and C. gattii VGII strains isolated previously from the environment and nasal cavities of koalas in park 4 (47) were therefore included in our analyses for comparative purposes, along with a disease isolate from another koala that contracted cryptococcosis while residing at park 4. Park 2 was closed in 2016, and most of its koalas were moved to parks 1 and 3, with a few transferred to external facilities.

The enclosure environment, including furniture and leaf browse, was similar across parks 1 to 3. All three parks also had similar protocols in place, enacted in March 2015, for the management of environmental C. gattii during this study. This included the changing of all koala perches every 6 months (more often if possible) and a decontamination protocol, enacted every 3 months (also more often if possible), during a “rest period” for each enclosure. This protocol included manual cleaning by scrubbing and hosing all surfaces with detergents followed by the dousing of the enclosure in a quaternary ammonium disinfectant (F10 SC; Health and Hygiene Pty Ltd., Roodepoort, South Africa) used at the manufacturer’s recommended concentration for fungal disinfection and utilizing a 30-min contact period. After this, enclosures were thoroughly rinsed with water, allowed to dry, and then eventually returned to use. Prior to this protocol, the facilities changed the perches less frequently (approximately every 12 months), and while the floors and walls of each enclosure were often scrubbed, less attention was paid to the furniture (perches, etc.) unless there was visible soiling.

### Koala nasal swabs.

Nasal swabs from parks 1 to 3 were submitted to Veterinary Pathology Diagnostic Services (VPDS), The University of Sydney, for culture. A sterile, moistened cotton-tipped swab was inserted into the nasal vestibule on both sides and rotated gently, as per methods of prior studies (16, 18). This procedure was performed by a veterinarian as part of a systematic disease control and management plan instigated at these facilities. In December 2015, 40 koalas were sampled at parks 1, 2, and 3 (7, 22, and 11 koalas at each park, respectively). In September 2017, 31 koalas were sampled at parks 1 and 3 (16 and 15 koalas, respectively). Across both sampling occasions, a total of 44 individuals were swabbed, with 27 swabbed on both occasions and 17 on one occasion only. The sampling of 17 koalas once only was attributable to either external transfers (koala no longer at any of the co-owned facilities or newly introduced in the time between the first and second samplings) or concerns regarding the stress of sampling from an individual (for example, mothers with young joeys).

### Environmental samples.

Samples were collected by moistening a sterile swab with sterile saline and running the tip thoroughly over the surface of perches and enclosure furniture, similar to previously described methods (16). In December 2015, samples were collected from all enclosures (16) at parks 1, 2, and 3 (4, 7, and 5 enclosures at each park, respectively). In September 2017, after the closure of park 2, 23 enclosures at parks 1 and 3 were sampled (13 and 10 at each park, respectively).

### Culture.

All swabs (koala nasal and environmental) were initially cultured on Staib’s bird seed agar containing antibiotics (penicillin and gentamicin) by rolling the swabs gently across the agar. Plates were incubated at 27°C and examined at least once daily for 7 to 10 days. Samples were considered positive if yeast-like colonies exhibiting the brown color effect (consistent with *Cryptococcus* spp.) were observed. If no growth was observed by 7 to 10 days, the plates were considered negative and discarded. Positive samples were classified according to the number of cryptococcal colonies counted on the agar plates as exhibiting either a low (1 to 10 colonies), moderate (11 to 100 colonies), or heavy (>100 colonies) extent of growth. A minimum of one cryptococcal colony from each positive plate was subcultured onto Sabouraud’s agar and incubated at 37°C for isolation and DNA extraction.

### Cryptococcal antigenemia testing.

Serum samples from 58 koalas were collected by veterinarians at the three facilities by cephalic venipuncture with the koalas gently restrained. Samples were collected at various time points between December 2014 and August 2018 as part of the ongoing disease investigation and surveillance program and submitted to VPDS. All samples underwent cryptococcal antigen testing using an LFA (CrAg LFA; IMMY, Norman, OK, USA). If the LFA was positive, an LCAT (CALAS; Meridian Bioscience, Inc., Cincinnati, OH, USA) was performed to confirm the result and establish a reciprocal antigen titer (36). Both procedures were performed according to the manufacturers’ instructions by experienced staff and in the same laboratory.

### Molecular and mating type determination.

DNA was extracted from all isolates using an established protocol for fungi (48). PCR amplification of the *URA5* gene was then performed, with the resulting product undergoing RFLP analysis and comparison to known standards (VGI, WM 179; VGII, WM 178; VGIII; WM 175; VGIV, WM 779; VNI, WM 148; VNII, WM 626; VNIII, WM 628; VNIV, WM 629) as described previously (49). This was to provide molecular confirmation that all isolates were C. gattii or C. neoformans and to identify the molecular type.

Determination of the mating type of all C. gattii VGII isolates was performed by PCR amplification of the *MF***a** and *MF*α genes and comparison to known standards (**a** standard, WM 06.38; α standard, WM 179) as described previously (50, 51). C. gattii VGI isolates did not proceed to mating type analysis.

### Multilocus sequence typing.

MLST of all C. gattii VGII isolates was performed according to the ISHAM consensus scheme for C. neoformans and C. gattii (52). This involved the PCR amplification and sequencing of seven loci: *CAP59*, *GPD1*, IGS1, *LAC1*, *PLB1*, *SOD1*, and *URA5*. The Fungal MLST database (http://mlst.mycologylab.org/) was then used to assign allele types and STs. C. gattii VGI isolates did not proceed to MLST analysis.

### Multilocus phylogenetic analysis.

Concatenated MLST sequences were aligned (MUSCLE), and a maximum likelihood phylogenetic analysis was performed (Kimura 2-parameter model [53] with gamma distribution) with 1,000 bootstrap replicates using the MEGA7 program (54). The following C. gattii standard strains were included for out grouping: WM 179 (VGI), WM 178 (VGII), WM 175 (VGIII), and WM 779 (VGIV).

### Whole-genome sequencing.

A representative group of 14 C. gattii VGII isolates collected in parks 1 to 3 (park 1, 2/14; park 2, 6/14; park 3, 6/14) were selected for WGS, including all four available disease isolates. Three isolates from park 4 (of the same multilocus sequence type as isolates from parks 1 to 3) were also included for comparative purposes (one environmental, one colonizing, and one disease isolate), making a total of 17 isolates.

DNA for WGS was extracted using the Quick-DNA Fungal/Bacterial Miniprep kit (Zymo Research, Irvine, CA, USA) according to the manufacturer’s instructions. Genomic DNA then was fragmented using a Q800R2 sonicator (QSonica, Newtown, CT, USA) to approximately 500 bp, and genome libraries were prepared for paired-end sequencing using the NEBNext Ultra II DNA Library Prep kit (New England BioLabs, Ipswich, MA, USA) and quantified using the SequalPrep Normalization Plate kit (Thermo Fisher Scientific, Waltham, MA, USA). Libraries were pooled and sequenced at 2 × 150 bp on a NextSeq (Illumina, Inc., San Diego, CA, USA).

### Genomic data analysis.

Read data from 19 C. gattii VGIIb genomes, 17 from the present study and 2 additional C. gattii VGIIb Australian genomes for comparison (WM 03.27 and WM 04.71; BioSamples SAMN02851029 and SAMN02851030, respectively) were included in data analysis. Reads were trimmed of adapter sequence and low-quality bases with Trimmomatic v0.32 (55). Parameters were set for sliding windows of 5 bp and required quality of 20. After quality trimming, no reads shorter than 65 bp were accepted.

The phylogenetic analyses of the whole-genome read data were conducted as described previously (42), with minor modifications. In short, we identified high-certainty SNPs in the samples using the NASP pipeline (v. 1.1.2) (56) against a *de novo* assembly created with read data from isolate WM 18.93 using SPAdes (v3.10.1) (57) with “careful” setting enabled. The pipeline was set to use BWA (v 0.7.15) (58) as the read aligner and GATK (3.7) (59) as the SNP caller. The pipeline also filtered out positions with coverage below 10×, those with base concordance below 90% among the aligned reads, and any positions that were not present in all samples of the set.

Phylogenetic analysis was conducted using MEGA7 (54). Tree structure was inferred using maximum parsimony and calculating a 95% consensus of 40 most parsimonious trees from the whole-genome SNP data of the 19 C. gattii VGIIb isolates (Fig. 4). The trees were rooted on the basal most branch, inferred from analysis using outgroup C. gattii standard strains (VGI WM179, VGII WM178, VGIII WM175, and VGIV WM779). As an extended phylogeny, we also identified whole-genome SNP differences across 32 genomes of VGIIb veterinary and environmental isolates (17 from the present study, 7 other Australian isolates, and 8 from North America) following the same procedure described above (Fig. 5, strains described in Table S2 in the supplemental material).

### Data availability.

Each unique MLST allele type was submitted to GenBank (accession numbers MK133807 to MK133825). WGS data are available as BioProject PRJNA524387. All C. gattii VGII isolates collected for this study are available in the Medical Mycology Culture Collection at The Westmead Institute for Medical Research in Westmead, New South Wales, Australia (accession numbers and strain information in Table S1).

10.1128/mSphere.00216-19.1 TABLE S1 List of Cryptococcus gattii species complex isolates with multilocus sequence typing data included in this study. Strain information, allele types, and sequence types are listed. Standard strains are included at the top of the table (grey rows). Strains are subsequently ordered by sequence type number, then culture collection number. Isolates in boldface font were included in whole-genome analyses. Download Table S1, PDF file, 0.3 MB. Copyright © 2019 Schmertmann et al. 2019 Schmertmann et al. This content is distributed under the terms of the Creative Commons Attribution 4.0 International license.

10.1128/mSphere.00216-19.2 TABLE S2 List of all genomes included in the phylogenetic analysis of Cryptococcus gattii VGIIb in Fig. 5. Download Table S2, PDF file, 0.2 MB. Copyright © 2019 Schmertmann et al. 2019 Schmertmann et al. This content is distributed under the terms of the Creative Commons Attribution 4.0 International license.
