# Supplementary material for: Jet-Setting Koalas Spread Cryptococcus gattii VGII in Australia
Source: mSphere. 2019 Jun 5;4(3):e00216-19. doi: 10.1128/mSphere.00216-19 (PMC6553553; doi:10.1128/mSphere.00216-19)
Supplement: TABLE S1 [file mSphere.00216-19-st001.pdf]

WM number	Species and molecular type	Year	Country (state)	Category	Park	Specific source	MT	Allele and sequence type numbers							
								CAP59	*GPD1*	*IGS1*	*LAC1*	*PLB1*	*SOD1*	*URA5*	*ST*
175	*C. gattii* VGIII	-	USA	Enviro	-	Eucalypt (standard)	α	18	18	14	3	6	28	19	60
178	*C. gattii* VGII	1991	Australia (NSW)	Disease	-	Human - lung (standard)	α	1	17	16	16	14	19	7	21
179	*C. gattii* VGI	1993	Australia (NSW)	Disease	-	Human - CSF (standard)	α	16	5	3	5	5	32	12	51
779	*C. gattii* VGIV	1995	South Africa	Disease	-	Cheetah (standard)	α	17	10	8	18	3	37	11	70
**03.27**	***C. gattii* VGII**	**1999**	**Australia (NT)**	**Enviro**	**-**	**Eucalypt**	**α**	**2**	**6**	**10**	**4**	**2**	**15**	**2**	**7**
**04.71**	***C. gattii* VGII**	**1991**	**Australia (NSW)**	**Disease**	**-**	**Cat - nasal**	**α**	**2**	**6**	**10**	**4**	**2**	**15**	**2**	**7**
09.153	*C. gattii* VGII	2009	Australia (WA)	Enviro	4	New enclosure	α	2	6	10	4	2	15	2	7
**09.154**	***C. gattii* VGII**	**2009**	**Australia (WA)**	**Enviro**	**4**	**New enclosure**	**α**	**2**	**6**	**10**	**4**	**2**	**15**	**2**	**7**
**09.155**	***C. gattii* VGII**	**2009**	**Australia (WA)**	**Colonising**	**4**	**Koala - nasal**	**α**	**2**	**6**	**10**	**4**	**2**	**15**	**2**	**7**
**09.156**	***C. gattii* VGII**	**2009**	**Australia (WA)**	**Colonising**	**4**	**Koala - nasal**	**α**	**2**	**6**	**10**	**4**	**2**	**15**	**2**	**7**
09.157	*C. gattii* VGII	2009	Australia (WA)	Colonising	4	Koala - nasal	α	2	6	10	4	2	15	2	7
09.158	*C. gattii* VGII	2009	Australia (WA)	Colonising	4	Koala - nasal	α	2	6	10	4	2	15	2	7
09.160	*C. gattii* VGII	2009	Australia (WA)	Colonising	4	Koala - nasal	α	2	6	10	4	2	15	2	7
09.161	*C. gattii* VGII	2009	Australia (WA)	Colonising	4	Koala - nasal	α	2	6	10	4	2	15	2	7
09.163	*C. gattii* VGII	2009	Australia (WA)	Enviro	4	Old enclosure	α	2	6	10	4	2	15	2	7
09.164	*C. gattii* VGII	2009	Australia (WA)	Enviro	4	Old enclosure	α	2	6	10	4	2	15	2	7
09.166	*C. gattii* VGII	2009	Australia (WA)	Enviro	4	Visitor area	α	2	6	10	4	2	15	2	7
09.167	*C. gattii* VGII	2009	Australia (WA)	Colonising	4	Koala - nasal	α	2	6	10	4	2	15	2	7
12.198	*C. gattii* VGII	2012	Australia (WA)	Colonising	4	Koala - nasal	α	2	6	10	4	2	15	2	7
12.200	*C. gattii* VGII	2012	Australia (WA)	Colonising	4	Koala - nasal	α	2	6	10	4	2	15	2	7
12.201	*C. gattii* VGII	2012	Australia (WA)	Colonising	4	Koala - nasal	α	2	6	10	4	2	15	2	7
12.204	*C. gattii* VGII	2012	Australia (WA)	Colonising	4	Koala - nasal	α	2	6	10	4	2	15	2	7
12.205	*C. gattii* VGII	2012	Australia (WA)	Colonising	4	Koala - nasal	α	2	6	10	4	2	15	2	7

12.206	*C. gattii* VGII	2012	Australia (WA)	Colonising	4	Koala - nasal	α	2	6	10	4	2	15	2	7
12.208	*C. gattii* VGII	2012	Australia (WA)	Colonising	4	Koala - nasal	α	2	6	10	4	2	15	2	7
12.211	*C. gattii* VGII	2012	Australia (WA)	Enviro	4	Main enclosure	α	2	6	10	4	2	15	2	7
12.212	*C. gattii* VGII	2012	Australia (WA)	Enviro	4	Main enclosure	α	2	6	10	4	2	15	2	7
12.214	*C. gattii* VGII	2012	Australia (WA)	Enviro	4	Nursery enclosure	α	2	6	10	4	2	15	2	7
12.215	*C. gattii* VGII	2012	Australia (WA)	Enviro	4	QLD enclosure	α	2	6	10	4	2	15	2	7
12.216	*C. gattii* VGII	2012	Australia (WA)	Enviro	4	QLD enclosure	α	2	6	10	4	2	15	2	7
12.217	*C. gattii* VGII	2012	Australia (WA)	Enviro	4	VIC enclosure	α	2	6	10	4	2	15	2	7
12.219	*C. gattii* VGII	2012	Australia (WA)	Enviro	4	VIC enclosure	α	2	6	10	4	2	15	2	7
13.222	*C. gattii* VGII	2013	Australia (WA)	Colonising	4	Koala - nasal	α	2	6	10	4	2	15	2	7
13.223	*C. gattii* VGII	2013	Australia (WA)	Colonising	4	Koala - nasal	α	2	6	10	4	2	15	2	7
13.244	*C. gattii* VGII	2013	Australia (WA)	Colonising	4	Koala - nasal	α	2	6	10	4	2	15	2	7
**13.373**	***C. gattii* VGII**	**2013**	**Australia (QLD)**	**Disease**	**2**	**Koala - thoracic mass**	**α**	**2**	**6**	**10**	**4**	**2**	**15**	**2**	**7**
**14.206**	***C. gattii* VGII**	**2014**	**Australia (QLD)**	**Disease**	**3**	**Koala - nasal mass**	**α**	**2**	**6**	**10**	**4**	**2**	**15**	**2**	**7**
**16.20**	***C. gattii* VGII**	**2005**	**Australia (QLD)**	**Disease**	**4**	**Koala - nasal mass**	**α**	**2**	**6**	**10**	**4**	**2**	**15**	**2**	**7**
**17.119**	***C. gattii* VGII**	**2016**	**Australia (QLD)**	**Disease**	**3**	**Koala - nasal discharge**	**α**	**2**	**6**	**10**	**4**	**2**	**15**	**2**	**7**
**17.120**	***C. gattii* VGII**	**2016**	**Australia (QLD)**	**Disease**	**3**	**Koala - lymph node**	**α**	**2**	**6**	**10**	**4**	**2**	**15**	**2**	**7**
**18.14**	***C. gattii* VGII**	**2016**	**Australia (QLD)**	**Colonising**	**2**	**Koala - nasal**	**α**	**2**	**6**	**10**	**4**	**2**	**15**	**2**	**7**
18.74	*C. gattii* VGII	2016	Australia (QLD)	Enviro	2	Enclosure KOA4	α	2	6	10	4	2	15	2	7
**18.76**	***C. gattii* VGII**	**2016**	**Australia (QLD)**	**Enviro**	**2**	**Enclosure KOA8**	**α**	**2**	**6**	**10**	**4**	**2**	**15**	**2**	**7**
18.78	*C. gattii* VGII	2016	Australia (QLD)	Enviro	2	Enclosure KOA8	α	2	6	10	4	2	15	2	7
**18.79**	***C. gattii* VGII**	**2016**	**Australia (QLD)**	**Enviro**	**2**	**Enclosure KOA5**	**α**	**2**	**6**	**10**	**4**	**2**	**15**	**2**	**7**
18.80	*C. gattii* VGII	2016	Australia (QLD)	Enviro	2	Enclosure KOA5	α	2	6	10	4	2	15	2	7
18.81	*C. gattii* VGII	2016	Australia (QLD)	Enviro	2	Enclosure KOA5	α	2	6	10	4	2	15	2	7
18.82	*C. gattii* VGII	2016	Australia (QLD)	Enviro	2	Enclosure KOA5	α	2	6	10	4	2	15	2	7
18.83	*C. gattii* VGII	2016	Australia (QLD)	Enviro	2	Enclosure KOA5	α	2	6	10	4	2	15	2	7
18.84	*C. gattii* VGII	2016	Australia (QLD)	Enviro	2	Enclosure KOA6	α	2	6	10	4	2	15	2	7
**18.92**	***C. gattii* VGII**	**2017**	**Australia (QLD)**	**Colonising**	**3**	**Koala - nasal**	**α**	**2**	**6**	**10**	**4**	**2**	**15**	**2**	**7**

**18.93**	***C. gattii* VGII**	**2017**	**Australia (QLD)**	**Colonising**	**3**	**Koala - nasal**	**α**	**2**	**6**	**10**	**4**	**2**	**15**	**2**	**7**
**18.94**	***C. gattii* VGII**	**2017**	**Australia (QLD)**	**Colonising**	**3**	**Koala - nasal**	**α**	**2**	**6**	**10**	**4**	**2**	**15**	**2**	**7**
**18.95**	***C. gattii* VGII**	**2017**	**Australia (QLD)**	**Colonising**	**1**	**Koala - nasal**	**α**	**2**	**6**	**10**	**4**	**2**	**15**	**2**	**7**
18.96	*C. gattii* VGII	2017	Australia (QLD)	Enviro	1	Group enclosure	α	2	6	10	4	2	15	2	7
**18.97**	***C. gattii* VGII**	**2017**	**Australia (QLD)**	**Enviro**	**1**	**Group enclosure**	**α**	**2**	**6**	**10**	**4**	**2**	**15**	**2**	**7**
18.98	*C. gattii* VGII	2017	Australia (QLD)	Enviro	3	Enclosure 1A	α	2	6	10	4	2	15	2	7
**18.99**	***C. gattii* VGII**	**2017**	**Australia (QLD)**	**Enviro**	**3**	**Enclosure 1A**	**α**	**2**	**6**	**10**	**4**	**2**	**15**	**2**	**7**
18.152	*C. gattii* VGII	2017	Australia (QLD)	Enviro	1	GD Enclosure	α	2	6	10	4	2	15	2	7
18.158	*C. gattii* VGII	2017	Australia (QLD)	Colonising	3	Koala - nasal	α	2	6	10	4	2	15	2	7
18.160	*C. gattii* VGII	2017	Australia (QLD)	Colonising	3	Koala - nasal	α	2	6	10	4	2	15	2	7
18.161	*C. gattii* VGII	2016	Australia (QLD)	Colonising	1	Koala - nasal	α	2	6	10	4	2	15	2	7
18.162	*C. gattii* VGII	2016	Australia (QLD)	Colonising	1	Koala - nasal	α	2	6	10	4	2	15	2	7
18.163	*C. gattii* VGII	2016	Australia (QLD)	Colonising	2	Koala - nasal	α	2	6	10	4	2	15	2	7
18.164	*C. gattii* VGII	2016	Australia (QLD)	Enviro	2	Enclosure KOA8	α	2	6	10	4	2	15	2	7
18.165	*C. gattii* VGII	2017	Australia (QLD)	Enviro	1	Group enclosure	α	2	6	10	4	2	15	2	7
09.165	*C. gattii* VGII	2009	Australia (WA)	Enviro	4	Old enclosure	**a**	14	21	27	28	27	46	2	38
09.152	*C. gattii* VGII	2009	Australia (WA)	Enviro	4	New enclosure	α	27	6	4	4	1	43	7	48
12.197	*C. gattii* VGII	2012	Australia (WA)	Colonising	4	Koala - nasal	α	27	6	4	4	1	43	7	48
12.213	*C. gattii* VGII	2012	Australia (WA)	Enviro	4	Nursery enclosure	α	27	6	4	4	1	43	7	48
**18.12**	***C. gattii* VGII**	**2016**	**Australia (QLD)**	**Colonising**	**2**	**Koala - nasal**	**α**	**2**	**6**	**10**	**4**	**2**	**15**	**56**	**539**
18.13	*C. gattii* VGII	2016	Australia (QLD)	Colonising	2	Koala - nasal	α	2	6	10	4	2	15	56	539

Enviro = environmental; MT = mating type; NSW = New South Wales; NT = Northern Territory, QLD = Queensland; ST=sequence type; VIC = Victoria; WA = Western Australia; WM = Westmead Mycology Culture Collection. Locations of Parks 1-4: Park 1 = 16°39'47.1"S 145°33'51.9"E; Park 2 = 16°45'28.9"S 145°39'46.4"E; Park 3 = 16°49'07.9"S 145°37'58.3"E); Park 4 = 31°50'03.6"S 115°57'01.2"
