# Supplementary material for: Jet-Setting Koalas Spread Cryptococcus gattii VGII in Australia
Source: mSphere. 2019 Jun 5;4(3):e00216-19. doi: 10.1128/mSphere.00216-19 (PMC6553553; doi:10.1128/mSphere.00216-19)
Supplement: TABLE S2 [file mSphere.00216-19-st002.pdf]

Strain	Location	Category	Specific source	Year	Reference	BioSample number
B7394	Washington, USA	Veterinary	Cat	2008	[1]	SAMN02851018
B8554	Oregon, USA	Veterinary	Dog	2008	[2]	SAMN02851020
B8828	Washington, USA	Veterinary	Porpoise	2010	[1]	SAMN02851021
B9157	Washington, USA	Veterinary	Horse	2011	[2]	SAMN02851022
B9552	Washington, USA	Veterinary	Porpoise	2011	[2]	SAMN02851023
B9563	Washington, USA	Veterinary	Porpoise	2011	[2]	SAMN02851024
B9758	British Columbia, Canada	Environmental	Soil	2002	[2]	SAMN02851026
B11567	Nova Scotia, Canada	Veterinary	Deer - brain lesion	2015	[1]	SAMN07738531
V6	Sydney, NSW, Australia	Veterinary	Cat - CNS	1992	[3]	SAMN02906888
V9	Coogee, NSW, Australia	Veterinary	Cat – lesion aspirate	1992	[3]	SAMN02906887
V26	Rosemeadow, NSW, Australia	Veterinary	Cat – nasal lesion	1996	[3]	SAMN02906889
WM 1008	Blacktown, NSW, Australia	Environmental	*Eucalyptus tereticornis*	2000	[4]*	Pending
WM 03.27	Arnhem Land, NT, Australia	Environmental	*Eucalyptus* spp.	1992	[2]	SAMN02851029
WM 04.71	Chiswick, NSW, Australia	Veterinary	Cat – nasal lesion	1991	[2]	SAMN02851030
WM 09.154	Caversham, WA, Australia	Environmental	Koala enclosure	2009	This study	Pending
WM 09.155	Caversham, WA, Australia	Veterinary	Koala – nasal swab	2009	Unpublished	Pending
WM 09.156	Caversham, WA, Australia	Veterinary	Koala – nasal swab	2009	This study	Pending
WM 13.373	Palm Cove, QLD, Australia	Veterinary	Koala – thoracic mass	2013	This study	Pending
WM 14.206	Kuranda, QLD, Australia	Veterinary	Koala – nasal mass	2014	This study	Pending
WM 16.20	Currumbin, QLD, Australia	Veterinary	Koala – nasal mass	2005	This study	Pending
WM 17.119	Kuranda, QLD, Australia	Veterinary	Koala – nasal discharge	2016	This study	Pending
WM 17.120	Kuranda, QLD, Australia	Veterinary	Koala – lymph node	2016	This study	Pending
WM 18.12	Palm Cove, QLD, Australia	Veterinary	Koala – nasal swab	2016	This study	Pending
WM 18.14	Palm Cove, QLD, Australia	Veterinary	Koala – nasal swab	2016	This study	Pending
WM 18.76	Palm Cove, QLD, Australia	Environmental	Koala enclosure	2016	This study	Pending
WM 18.79	Palm Cove, QLD, Australia	Environmental	Koala enclosure	2016	This study	Pending
WM 18.92	Kuranda, QLD, Australia	Veterinary	Koala – nasal swab	2017	This study	Pending
WM 18.93	Kuranda, QLD, Australia	Veterinary	Koala – nasal swab	2017	This study	Pending
WM 18.94	Kuranda, QLD, Australia	Veterinary	Koala – nasal swab	2017	This study	Pending
WM 18.95	Wangetti, QLD, Australia	Veterinary	Koala – nasal swab	2017	This study	Pending
WM 18.97	Wangetti, QLD, Australia	Environmental	Koala enclosure	2017	This study	Pending
WM 18.99	Kuranda, QLD, Australia	Environmental	Koala enclosure	2017	This study	Pending

CNS = central nervous system; NSW = New South Wales; NT = Northern Territory; QLD = Queensland; WA = Western Australia.

*Genome unpublished. Reference acknowledges the source of initial isolation.

[1] Roe CC, Bowers J, Oltean H, et al. Dating the *Cryptococcus gattii* dispersal to the North American Pacific Northwest. *mSphere.* 2018; 3 (1): e00499-00417

[2] Engelthaler DM, Hicks ND, Gillece JD, et al. *Cryptococcus gattii* in North American Pacific Northwest: whole-population genome analysis provides insights into species evolution and dispersal. *mBio.* 2014; 5 (4): e01464-01414.

[3] Billmyre RB, Croll D, Li W, et al. Highly recombinant VGII *Cryptococcus gattii* population develops clonal outbreak clusters through both sexual macroevolution and asexual microevolution. *mBio.* 2014; 5 (4): e01494-01414

[4] Kidd SE, Sorrell TC, Meyer W. Isolation of two molecular types of *Cryptococcus neoformans* var. *gattii* from insect frass. *Med Mycol.* 2003; 41 (2): 171-176.
